# Association between oral health and locomotive syndrome: a cross-sectional study in Japanese adults

**DOI:** 10.1186/s12877-023-04572-z

**Published:** 2023-12-13

**Authors:** Mizuki Saito, Yoshihiro Shimazaki, Saori Yoshii, Hideo Takeyama

**Affiliations:** 1 Department of Preventive Dentistry and Dental Public Health, School of Dentistry, Aichi Gakuin University, 1-100 Kusumoto-cho, Chikusa-ku, Nagoya, 464-8650 Japan; 2 Aichi Health Promotion Foundation, Nagoya, Japan

**Keywords:** Locomotive syndrome, Chewing function, Tooth loss

## Abstract

**Background:**

Many studies have examined the association between oral health, care needs, and physical function, but few have focused on the association between oral health and locomotive syndrome (LS). We examined the association between LS and oral-health status, such as the number of teeth and chewing function, in an adult population.

**Methods:**

The study included 2888 participants who underwent examinations of motor function and oral health. Individuals with LS stage 1 or higher were classified as having LS, while others were classified as not having it. Logistic regression analysis was performed using the presence or absence of LS as the dependent variable and age, sex, smoking status, drinking habit, exercise habit, walking speed, history of stroke, bone density, body mass index, metabolic syndrome, chewing function, and the number of teeth as independent variables to calculate the odds ratios (ORs) and 95% confidence intervals (CIs) for each independent variable.

**Results:**

When the number of teeth and chewing function were included separately in multivariate analyses, the OR for LS was significantly higher for participants with 0–19 teeth than for those with 28 teeth, and for participants with poor chewing function than for those with good function (adjusted ORs, 1.47 [95% CI, 1.01–2.15] and 1.73 [95% CI, 1.37–2.18], respectively). In analyses that included tooth number and chewing function as a combined independent variable, relative to individuals with 28 teeth and good masticatory function, the adjusted ORs were 2.67 (95% CI, 1.57–4.52) for those with 28 teeth and poor chewing function, 1.63 (95% CI, 1.20–2.22) for those with 20–27 teeth and poor chewing function, and 1.83 (95% CI, 1.06–3.18) for those with 0–19 teeth and poor chewing function.

**Conclusion:**

Having fewer teeth and poor chewing function may be associated with LS. The maintenance of masticatory function may be important to prevent LS in adulthood.

## Background

Decline in physiological reserves due to organ dysfunction leads to a state of frailty in older people that can lead to outcomes such as functional disability, need for nursing care, and death [1]. Locomotive syndrome (LS) is a condition in which mobility is impaired due to age-related muscle weakness and disorders of the locomotor system, such as bone, nerve, muscle, and joint diseases. It is estimated that 47 million people in Japan have LS, including those with preexisting conditions [2], and the number is likely to increase as the population ages.

Social participation and the activities of daily living can be limited by LS [3]. Thus, LS affects both physical- and mental-health status, such as through a lack of exercise and staying at home, and significantly reduces the quality of life of older individuals [4]. In addition, more than 20% of the need for long-term care in older people is due to motor-system disorders, such as joint diseases, fractures, and falls [5]. As LS is a risk factor for conditions requiring long-term care, its prevention is important from the perspective of avoiding the need for long-term care. Notably, muscle weakness tends to start in middle age and progress with age [6]; thus, the risk for LS should be considered not only in the older people but also in the middle-aged adult population.

Loss of teeth and chewing dysfunction are associated with reduced mobility and risk for functional disability [7–9]. In a cohort study of older people aged ≥60 years, those with tooth loss at baseline were slower walkers compared to those without tooth loss [7]. In a study of older people in the UK, as the number of remaining natural teeth increased, the probability of the instrumental activities of daily life being limited decreased [8]. In addition, individuals with reduced chewing function may have less independence [9]. The loss of many teeth and poor chewing function are associated with an unbalanced diet and malnutrition [10], which negatively affect physical function [11]. Thus, while many studies have assessed the relationship between oral health, care needs, and physical functioning, few have investigated the association between oral-health status and LS.

We examined the association between LS and oral-health status, such as the number of teeth and chewing function, in an adult population.

## Methods

### Participants

In total, 12,922 individuals had oral examinations as part of general checkups performed at the Aichi Health Promotion Foundation between April 1, 2018, and March 31, 2019. Of these, motor function was also examined in 5230 individuals. Participants in this study were aged ≥50 years. In total, 2888 individuals with no missing data were included. The mean age of the participants was 60.15 (standard deviation, 7.69; range, 50–94) years.

### Oral health examination

An oral-health examination was conducted to evaluate the condition of the teeth, recorded as sound, decayed, filled, or missing. The total number of teeth (excluding third molars) was used as the number of teeth. Residual tooth roots and root caps were considered decayed and filled teeth, respectively, and were included in the number of teeth.

### Systemic health examination

Each participant’s height and weight were measured, and their body mass index (BMI) was calculated. Waist circumference, blood pressure, fasting blood glucose, and blood lipids (triglycerides and high-density lipoprotein cholesterol) were also measured, and metabolic syndrome was assessed from these results based on the Japanese criteria [12]. Individuals who met one item, in addition to having a large waist circumference, were classified as having pre-metabolic syndrome, and those who met two or more items other than a large waist circumference were classified as having metabolic syndrome. Individuals under treatment for, or taking medication related to, the components of metabolic syndrome were considered positive for the respective components.

### Determination of locomotive syndrome

The criteria proposed by the Japanese Orthopedic Association were used to determine LS [13]. Judgments were made based on the results of the stand-up test, the two-step test, and the 25-question Geriatric Locomotive Function Scale (GLFS-25). The stand-up test involves participants sitting on a 40-, 30-, 20-, or 10-cm platform and determining whether they are able to stand up using both legs or one leg. If a person is unable to stand up from a 30-cm platform using both legs, the degree of LS is stage 3; if a person cannot stand up from a 20-cm platform using both legs but can stand up from a 30-cm platform, the degree of LS is stage 2; if a person cannot stand up from a 40-cm platform using one leg but can stand up from a 20-cm platform using both legs, the degree of LS is stage 1. In the two-step test, the individual walks two steps with the thighs as far apart as possible, and the value obtained by dividing their stride length by their height is the two-step value. Stage 3 LS is defined as two-step values < 0.9, stage 2 by values of 0.9 to < 1.1, and stage 1 by values of 1.1 to < 1.3. The GLFS-25 consists of 25 questions about physical and living conditions. It is rated on a scale from 0 to 100, with higher scores indicating more advanced LS. A score ≥ 24 indicates LS stage 3, a score of 16–23 indicates LS stage 2, and a score of 7–15 indicates LS stage 1. Of the LS stages based on the results of each test, the highest stage was used as the individual’s LS stage.

### Questionnaire

A self-administered questionnaire was used to investigate smoking status (never/past/current), drinking habit (no/sometimes/everyday), exercise habit, walking speed, chewing function, and history of stroke. Participants were asked “Have you engaged in light, sweaty exercise for at least 30 min at a time, at least twice per week, for at least 1 year?” The respondents were asked about their walking speed. “Do you walk faster than your peers of approximately the same age?” Regarding chewing function, “Which of the following applies to your condition when you chew your food?” and they answered from three options: “I can chew and eat anything,” “I have some concerns about my teeth, gums, and bite, and sometimes have difficulty chewing,” and “I can hardly chew.”

### Statistical analysis

Most participants in this study retained more than 20 teeth. Therefore, numbers of teeth were categorized as 0–19, 20–27, and 28 [14]. For chewing function, those who responded “I can chew and eat anything” were defined as the “good” group, and those who responded “I have some concerns about my teeth, gums, and bite, and sometimes have difficulty chewing” or “I can hardly chew” were defined as the “poor” group. Individuals with LS stage 1 or higher were classified as patients with LS, while others were classified as patients without LS.

The chi-square test was used to examine differences in the proportions of categorical variables and the unpaired *t*-test was used to examine differences in the mean values of continuous variables according to the presence or absence of LS. Logistic regression analysis was performed using the presence or absence of LS as the dependent variable and age, sex, smoking status, drinking habit, exercise habit, walking speed, history of stroke, bone mineral density, BMI, metabolic syndrome, chewing function, and number of teeth as independent variables to calculate the odds ratios (ORs) and 95% confidence intervals (CIs) for each independent variable. The associations between the number of teeth and chewing function and LS were also examined, as one study found a strong association between the number of teeth and chewing function [15]. Therefore, we checked for multicollinearity among these variables using the variance inflation factor (VIF). All statistical analyses were performed with SPSS ver. 26.0 software (IBM Corp., Armonk, NY, USA). A *P-*value < 0.05 was considered significant.

## Results

The characteristics of participants according to LS status are shown in Table 1. Of the participants, 856 (29.6%) had LS. Significant differences in age, BMI, bone mineral density, sex, drinking habit, exercise habit, walking speed, metabolic syndrome, chewing function, and number of teeth were observed between participants with and without LS.

**Table 1:** Associations between the characteristics of the participants and locomotive syndrome

Variable	Locomotive syndrome		*P*–value
	Absence (*N* = 2032)	Presence (*N* = 856)	
	Mean (SD)		
Age	58.96 (6.88)	62.98 (8.72)	< 0.001
Body mass index (kg/m^2^)	23.40 (3.17)	23.86 (3.62)	< 0.001
Bone mineral density	2.65 (0.31)	2.59 (0.32)	< 0.001
	N (%)		
Sex			
Men	1535 (75.5)	563 (65.8)	< 0.001
Women	497 (24.5)	293 (34.2)	
Smoking status			
Never	942 (46.4)	424 (49.5)	0.088
Past	688 (33.9)	281 (32.8)	
Current	402 (19.8)	151 (17.6)	
Drinking habit			
No	565 (27.8)	307 (35.9)	< 0.001
Sometimes	689 (33.9)	276 (32.2)	
Everyday	778 (38.3)	273 (31.9)	
Exercise habit			
Yes	571 (28.1)	193 (22.5)	0.002
No	1461 (71.9)	663 (77.5)	
Walking speed			
Fast	804 (39.6)	217 (25.4)	< 0.001
Slow	1228 (60.4)	639 (74.6)	
Stroke (past history)			
No	2004 (98.6)	841 (98.2)	0.448
Yes	28 (1.4)	15 (1.8)	
Metabolic syndrome			
No	1349 (66.4)	509 (59.5)	< 0.001
Pre-metabolic syndrome	311 (15.3)	136 (15.9)	
Metabolic syndrome	372 (18.3)	211 (24.6)	
Chewing function			
Good	1799 (88.5)	686 (80.1)	< 0.001
Poor	233 (11.5)	170 (19.9)	
Number of teeth			
28 teeth	788 (38.8)	260 (30.4)	< 0.001
20–27 teeth	1158 (57.0)	517 (60.4)	
0–19 teeth	86 (4.2)	79 (9.2)	

*SD* standard deviation

Table 2 shows the results of logistic regression analysis in which the presence or absence of LS was used as the dependent variable and the number of teeth and chewing function were simultaneously or separately entered as independent variables. The VIF for tooth number and chewing function for locomotion was 1.05, a low value indicating that multicollinearity was not a problem.

**Table 2:** Logistic regression analysis of the associations between the independent variables and locomotive syndrome

Independent variable	Dependent variable: locomotive syndrome (absence = 0, presence = 1)							
	Crude OR (95% CI)	*P*-value	Adjusted OR^a^ (95% CI)	*P*-value	Adjusted OR^b^ (95% CI)	*P*-value	Adjusted OR^c^ (95% CI)	*P*-value
Age	1.07 (1.06–1.08)	< 0.001	1.07 (1.06–1.09)	< 0.001	1.07 (1.06–1.09)	< 0.001	1.07 (1.06–1.09)	< 0.001
Body mass index (kg/m^2^)	1.04 (1.02–1.07)	< 0.001	1.06 (1.02–1.09)	< 0.001	1.06 (1.02–1.09)	< 0.001	1.06 (1.02–1.09)	< 0.001
Bone mineral density	0.50 (0.38–0.65)	< 0.001	0.74 (0.54–1.01)	0.061	0.73 (0.54–1.01)	0.056	0.74 (0.54–1.01)	0.060
Sex								
Men	1		1		1		1	
Women	1.61 (1.35–1.91)	< 0.001	1.76 (1.37–2.26)	< 0.001	1.76 (1.37–2.26)	< 0.001	1.76 (1.37–2.26)	< 0.001
Smoking status								
Never	1		1		1		1	
Past	0.91 (0.76–1.09)	0.290	1.06 (0.85–1.33)	0.590	1.07 (0.86–1.33)	0.564	1.07 (0.86–1.33)	0.555
Current	0.84 (0.67–1.04)	0.106	1.19 (0.92–1.56)	0.191	1.24 (0.95–1.61)	0.114	1.21 (0.93–1.58)	0.154
Drinking habit								
No	1		1		1		1	
Sometimes	0.74 (0.61–0.90)	0.002	0.95 (0.77–1.18)	0.670	0.95 (0.76–1.17)	0.619	0.95 (0.77–1.18)	0.657
Everyday	0.65 (0.53–0.79)	< 0.001	0.79 (0.64–0.99)	0.042	0.80 (0.64–1.00)	0.052	0.79 (0.64–0.99)	0.041
Exercise habit								
Yes	1		1		1		1	
No	1.34 (1.11–1.62)	0.002	1.49 (1.22–1.83)	< 0.001	1.50 (1.23–1.83)	< 0.001	1.50 (1.22–1.83)	< 0.001
Walking speed								
Fast	1		1		1		1	
Slow	1.93 (1.61–2.30)	< 0.001	1.95 (1.61–2.35)	< 0.001	1.98 (1.64–2.38)	< 0.001	1.94 (1.61–2.34)	< 0.001
Metabolic syndrome								
No	1		1		1		1	
Pre-metabolic syndrome	1.16 (0.93–1.45)	0.200	1.15 (0.87–1.51)	0.336	1.16 (0.88–1.53)	0.292	1.15 (0.87–1.51)	0.336
Metabolic syndrome	1.50 (1.23–1.83)	< 0.001	1.24 (0.95–1.62)	0.112	1.24 (0.96–1.62)	0.106	1.24 (0.95–1.62)	0.108
Chewing function					–			
Good	1		1				1	
Poor	1.91 (1.54–2.38)	< 0.001	1.69 (1.33–2.14)	< 0.001			1.73 (1.37–2.18)	< 0.001
Number of teeth							–	
28 teeth	1		1		1			
20–27 teeth	1.35 (1.14–1.61)	< 0.001	0.99 (0.82–1.20)	0.910	1.04 (0.86–1.26)	0.694		
0–19 teeth	2.78 (1.99–3.90)	< 0.001	1.25 (0.85–1.83)	0.265	1.47 (1.01–2.15)	0.044		

*OR* odds ratio, *CI* confidence interval

^a^ Adjusted model includes age, sex, smoking status, drinking habit, exercise habit, walking speed, body mass index, bone mineral density, metabolic syndrome, chewing function, and number of teeth

^b^ Adjusted model includes age, sex, smoking status, drinking habit, exercise habit, walking speed, body mass index, bone mineral density, metabolic syndrome, and number of teeth

^c^ Adjusted model includes age, sex, smoking status, drinking habit, exercise habit, walking speed, body mass index, bone mineral density, metabolic syndrome, and chewing function

In multivariate analysis of these factors together, age, BMI, sex, drinking habit, exercise habit, walking speed, and chewing function were significantly associated with LS. Participants with chewing difficulties had a significantly higher OR for LS compared to those with good chewing function (adjusted OR, 1.69 [95% CI, 1.33–2.14]). No significant association was detected between the number of teeth and LS. In analyses considering each of these factors separately, each was significantly associated with LS. The OR for LS was significantly higher in participants with 0–19 teeth than in those with 28 teeth (adjusted OR, 1.47 [95% CI, 1.01–2.15]). Participants with poor chewing function had a significantly higher OR for LS than those with good chewing function [adjusted OR, 1.73 [95% CI, 1.37–2.18]).

Table 3 shows the results of the analysis including the number of teeth and chewing function as a combined independent variable. Relative to individuals with 28 teeth and good masticatory function, the adjusted OR for those with 28 teeth and poor chewing function was 2.67 (95% CI, 1.57–4.52); for those with 20–27 teeth and poor chewing function, 1.63 (95% CI, 1.20–2.22); and for those with 0–19 teeth and poor chewing function, 1.83 (95% CI, 1.06–3.18).

**Table 3:** Logistic regression analysis of the association between the combined variables of the number of teeth and chewing function and locomotive syndrome

Independent variable	N (%)	Dependent variable: locomotive syndrome (absence = 0, presence = 1)			
		Crude OR (95% CI)	*P* value	Adjusted OR^a^ (95% CI)	*P*-value
Number of teeth / Chewing function					
28 teeth / Good	980 (33.9)	1		1	
20–27 teeth / Good	1408 (48.8)	1.37 (1.14–1.65)	0.001	1.04 (0.85–1.28)	0.691
0–19 teeth / Good	97 (3.4)	2.85 (1.87–4.37)	< 0.001	1.46 (0.92–2.33)	0.110
28 teeth / Poor	68 (2.4)	2.93 (1.78–4.83)	< 0.001	2.67 (1.57–4.52)	< 0.001
20–27 teeth / Poor	267 (9.2)	2.10 (1.58–2.80)	< 0.001	1.63 (1.20–2.22)	0.002
0–19 teeth / Poor	68 (2.4)	3.30 (2.01–5.43)	< 0.001	1.83 (1.06–3.18)	0.031

*OR* odds ratio, *CI* confidence interval

^a^ Adjusted for age, sex, smoking status, drinking habit, exercise habit, walking speed, body mass index, bone mineral density, and metabolic syndrome

## Discussion

In this analysis of the association between oral-health status and LS in an adult population, having fewer teeth and poor chewing function were significantly associated with LS. However, in analyses that considered these factors simultaneously, only the association with chewing function remained, indicating that LS is more closely related with chewing function than with number of teeth.

Tooth loss is a risk factor for reduced mobility, such as walking speed and stair climbing [16, 17]. The loss of many teeth also increases the need for long-term care [18]. Older people with poor self-evaluated chewing function show lower activity and less ability to engage in daily living activities [9]. Tooth loss and the accompanying decline in chewing function present the risk of malnutrition [19]. Malnutrition in older people can lead to frailty, sarcopenia, and mortality. In this study, individuals with many missing teeth or poor chewing function had worse nutritional status, which may have led to LS. In addition, tooth loss is associated with locomotor diseases that cause LS, including osteoporosis and rheumatoid arthritis [20–22], which may increase the risk for LS onset. Therefore, those with fewer teeth may be at higher risk for LS.

In this study, analyses that included both tooth number and chewing function simultaneously as independent variables showed that LS was significantly associated only with the latter. In analyses that combined these factors, the risk for LS was significantly higher among those with 28 teeth but with reduced chewing function, implying that chewing function has a stronger effect on LS than does number of teeth. As chewing function is closely related to number of teeth, its restoration in individuals with many lost teeth through prosthetic treatment may be important to prevent LS. On the other hand, people with many teeth but poor chewing function were at greater risk for LS. However, chewing function has been reported to be influenced not only by the number of teeth and periodontal condition but also by the masticatory muscles [23]. The subjects in our study who reported not being able to chew well despite having a full set of teeth were more likely to have decreased masticatory muscle strength than were those with few teeth and difficulty in chewing. A significant correlation has been found between masseter muscle thickness and the skeletal muscle index [24], and people with weak masseter muscles may also have weak skeletal muscles throughout the body. Some reports have suggested a link between sarcopenia and decreased masticatory function [25, 26]. Sarcopenia is a condition of decreased skeletal-muscle mass and muscle weakness throughout the body and is a cause of LS. Thus, those with 28 teeth and poor chewing function would have the highest OR for LS. Training to recover masticatory function is important in this population.

Clenching the teeth affects postural stability. Afferent impulses induced by clenching promote the H reflex in the soleus and tibialis anterior muscles, the antigravity muscles of the lower limbs. This occurs via sensory receptors in the periodontal ligament and muscle spindles of the masticatory muscles to the cerebral cortex [27], thereby contributing to postural stability. When in an upright position, frequent small oscillations are generated in the body to maintain balance. A stable jaw position stabilizes the contraction of the sternocleidomastoid and reduces general body sway [28]. Therefore, decreased support from teeth and occlusion leads to deterioration in leg strength and balance. In a cross-sectional study of older Japanese, participants with more occlusal support had higher dynamic strength in their lower extremities and longer open-eyed one-leg standing times than those who did not [29]. Loss of teeth and less occlusal support may decrease muscle strength and balance in the lower extremities, leading to LS. If dental treatment can restore the occlusal state and prevent LS, it may reduce the risk for frailty and the need for long-term care, leading to an extended life expectancy.

Several limitations of this study should be discussed. Because we used a cross-sectional design, a causal relationship between number of teeth and chewing function and LS cannot be claimed. Chewing function was based on self-reports and may have differed from objective chewing function [30]. Psychological factors such as depression have also been associated with subjective assessment of chewing function [31, 32]. In addition, a relationship between depression and LS has been reported, but we did not consider such factors in this study [33]. Our results may not be generalizable because the participants were limited to a specific area of Japan.

In conclusion, people with fewer teeth and poor chewing function are at significantly greater risk for LS. Prevention of tooth loss through appropriate self-care and professional care, evaluation of oral function, and training to prevent deterioration of oral function help to prevent LS in old age.

## Data Availability

The data that support the findings of this study are available from the Aichi Health Promotion Foundation, but restrictions apply to the availability of these data, which were used under license for the current study, and so are not publicly available. Data are however available from the authors upon reasonable request and with permission of the Aichi Health Promotion Foundation.

## Abbreviations

LS: locomotive syndrome
BMI: body mass index
OR: odds ratio
CI: confidence interval
